# Properties of Polymer-Modified Cement–Water-Glass Slurry

**DOI:** 10.3390/ma17163888

**Published:** 2024-08-06

**Authors:** Qian Yang, Junxiang Xu, Yiheng Ju, Dewang Lu, Wei Meng, Jing Wu, Xuefu Zhang

**Affiliations:** 1 The Fourth Engineering Co., Ltd. of CCCC First Highway Engineering Co., Ltd., Nanning 530031, China; 13060061625@163.com (Q.Y.); 13028317521@163.com (D.L.); wujing63211@163.com (J.W.); 2 School of Civil Engineering, Chongqing Jiaotong University, Chongqing 400074, China; xujuxiang6321@163.com (J.X.); 15330350334@163.com (Y.J.); zhangxuefu6321@163.com (X.Z.)

**Keywords:** polyurethane, acrylic acid, dual-liquid slurry performance

## Abstract

The corrosion resistance of cement–water-glass dual-liquid slurry is poor. Improving its material properties is necessary. In this study, we examined the influence of water-based lotions on the fluidity, gelling time, and mechanical properties of a cement–water-glass dual-liquid slurry based on the mix proportion of the dual-liquid slurry commonly used in construction. The mixture ratio of a C-S (cement–water-glass slurry) dual-liquid slurry was adjusted by introducing a waterborne polyurethane lotion and a waterborne acrylic lotion to modify the traditional C-S dual-liquid slurry material. When acrylic acid is used as a modifying polymer at a dosage of 7.5%, the flowability and gelation time of the dual-liquid slurry are excellent, the compressive strength of the stone body decreases slightly, the flexural strength is improved to a certain extent, and the stone body’s crack resistance during water loss is also enhanced. Moreover, the porosity of the stone body is low.

## 1. Introduction

### 1.1. Research Background

Since its establishment, the People’s Republic of China has achieved many remarkable results in the field of transportation construction. However, the rapid development of transportation construction has also resulted in a series of resource, environmental, and ecological difficulties. The deepening understanding of sustainable development and the development plan of national green transportation from the 14th Five-Year Plan of China have resulted in increasingly advanced requirements for transportation infrastructure construction, especially tunnel construction. In this context, how to construct high-quality tunnels has become a widely discussed topic [1,2].

Water is an important factor that cannot be ignored in tunnel construction, and water leakage is prevalent. Grouting is often used to reinforce and control fractured rock masses during tunnel construction. Static pressure grouting or high-pressure jet grouting is frequently used to inject preconfigured permeable grout into a rock mass to strengthen the rock and soil, treat diseases or geological disasters, and improve the geological engineering conditions [3,4].

**Figure 1:**
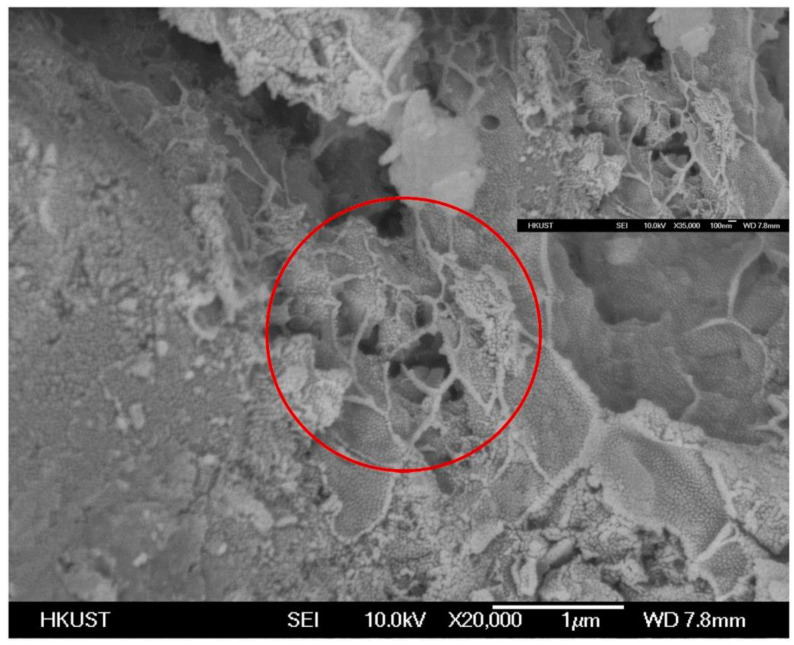
Example of the interweaving and adsorption of a polymer membrane structure [15].

**Figure 2:**
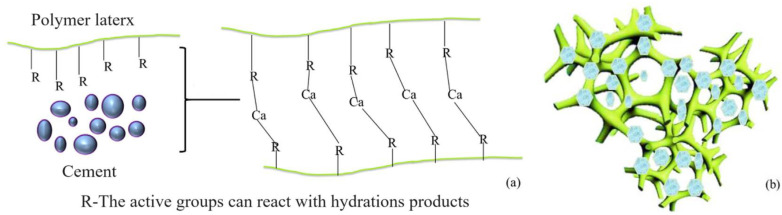
(**a**) Chemical reactions between polymers and hydration products; (**b**) three-dimensional network structure of modified polymer cement system [12].

Cement–water-glass dual-liquid slurry, also known as C-S slurry, has characteristics such as a short cementation time, a high initial stone strength, and an extremely high stone-creation rate. A mixture of cement slurry (C-liquid) and water glass (S-liquid) injected into underground rock layers or soil can penetrate into cracks, pores, and voids. This changes the flow and permeability characteristics of the groundwater to control it, thus impeding water. However, issues such as poor durability, significant dry shrinkage, and groundwater pollution render it necessary to improve the performance of the materials to meet engineering requirements [5,6,7]. The modification of cement–water-glass double-liquid slurry using a polymer lotion mainly involves a reaction between the polymer and cement. Common polymers used for modifying cement-based materials include epoxy resin, polyurethane, and acrylic acid [8]. An epoxy lotion needs to be used with a curing agent in cement-based materials, and common curing agents have compatibility problems with water glass, so they are not suitable for modifying double-liquid slurry materials with a large water–cement ratio and strong alkalinity [9,10]. A waterborne polyurethane lotion is a high molecular-weight polymer lotion in which water is the dispersion medium. It has no free isocyanate, and its molecular structure contains a carbamate group (-NH-COO-). A waterborne acrylic lotion is a lotion copolymerized by pure acrylate monomers, containing carboxyl (-COOH); it is cheap, safe, and environmentally friendly, and it has good water and alkali resistance. Many scholars have studied the modification mechanisms of polymer-modified cement-based materials containing ester and amino functional groups [11,12,13]. Wang et al. [14] analyzed the influence of styrene acrylic latex on a cement hydration process using an infrared spectrum and a scanning microscope. They observed that a significant amount of COOH was separated into COO^−^ and H^+^ in a rich alkali solution. The generated COO^−^ combined with Ca^2+^, Al^3+^, and other metal cations in the hydration product through an ionic bond to form a compound with a macromolecular filamentous network structure. In the red marked section, the polymer network is interconnected through strong interactions or hydration between inorganic silicate chains and organic PAAS chains. This was adsorbed on the surface of silicate, as demonstrated in Figure 1. The reaction equation is presented below, and the schematic diagram is provided in Figure 2.

R-COOH→R-COO^−^ + H^+^ (1)

2R-COO^−^ + Ca^2+^→R-COO·Ca·OOC-R (2)

Na^+^ and K^+^ plasmas can also connect with oxygen atoms. Salt bridges enhance interface bonding, suppress crack development, and improve the ductility of cement–polymer composites.

In a water-rich environment, substances such as NaOH in the stone body easily dissolve. This leads to an increase in alkalinity and a deterioration in the stability of C-S-H gels and other substances, resulting in structural damage to the stone body. There may also be water glass in a dual-liquid slurry stone body; this does not participate in the hydration reaction of a dissolved water solution and can lead to the decline or even failure of the water-blocking performance of the stone body over time [15]. Both the cement and water glass components in cement–water-glass dual-liquid slurry material have a degree of solubility. Although cement–water-glass dual-liquid slurry is a common slurry material in engineering, there is scant research on the durability of C-S dual-liquid slurry material in China. From the perspective of a sustainable development strategy, researching improvements in the corrosion resistance of dual-liquid slurry material is significant and valuable for engineering construction, disaster prevention and control, and ecological environment protection.

### 1.2. Research Content

In this study, we discussed the basic working performance of cement–water-glass dual-liquid slurry material. We selected the common construction mix proportion of dual-liquid slurry as the basic mix proportion and used a modification to a polymer lotion to study the influence of the polymer lotion on the performance of traditional dual-liquid slurry. Performance testing and characterization analyses of two types of polymer-modified dual-liquid slurries were conducted, and we selected the polymer and dosage that were most suitable for the dual-liquid slurry material.

## 2. Test Materials and Instruments

### 2.1. Test Materials

In addition to using common cement, water glass (Bomei degree 39Bé, density 1.373 g/cm^3^, Na_2_O 8.35%, SiO_2_ 27.41%, Modulus 3.21), and water, the following experimental materials were also used in this experiment.

1. Retarder

The retarder used in this experiment was analytical pure disodium hydrogen phosphate, which was produced by Kemio Chemical Reagent Co., Ltd., Tianjin, China. It had a purity of ≥99%, a molecular formula of Na_2_HPO_4_·12H_2_O, and a molecular weight of 358.1. It complied with the Technical Specification for the Application of Concrete Admixtures (GB50119-2013) standard [16].

2. Waterborne polyurethane

**Figure 3:**
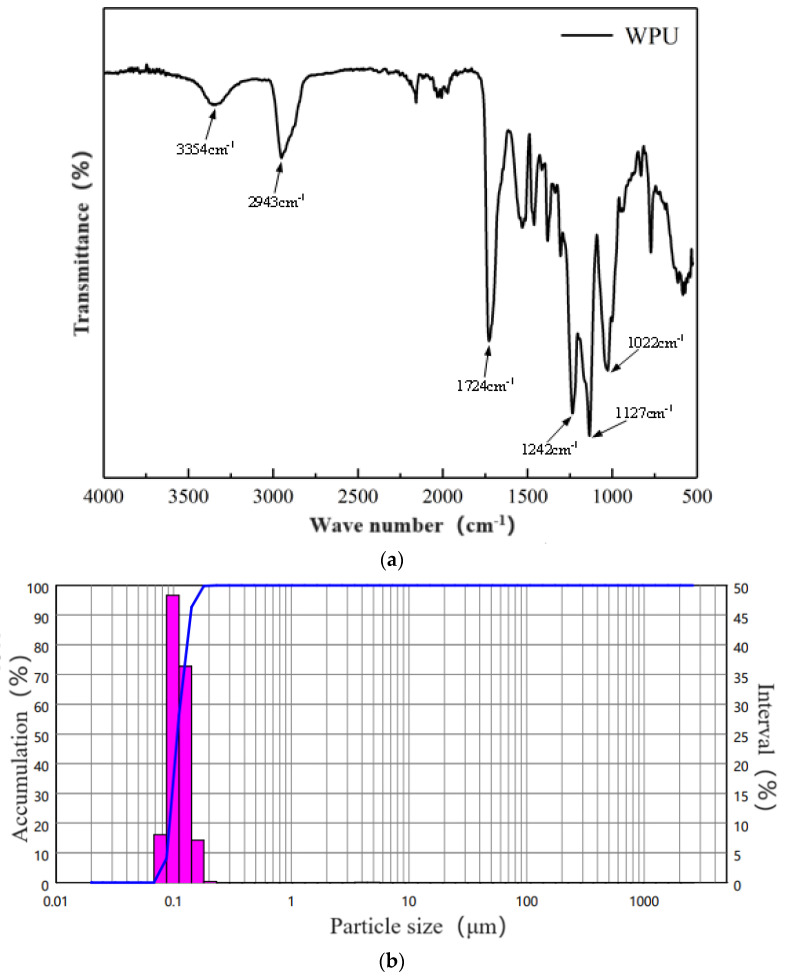
(**a**) Infrared spectrum of waterborne polyurethane lotion. (**b**) Particle size curve of waterborne polyurethane (The red part represents the particle size distribution of the material, and the blue part represents the cumulative amount of each interval).

The polyurethane used in this test was PU-601R, a waterborne polyurethane lotion produced by Hefei Huayue New Material Technology Co., Ltd., Hefei, China. The infrared spectrum obtained using a Brooke Vertex 70 (Bruker Optics, Shanghai, China) is presented in Figure 3 (WPU: waterborne polyurethane). The -NH stretching vibration peak corresponded with a wavelength of 3354 cm^−1^, the -CH_2_- stretching vibration peak corresponded with a wavelength of 2943 cm^−1^, the C=O stretching vibration peak corresponded with a wavelength of 1724 cm^−1^, the C-N stretching vibration peak corresponded with wavelengths of 1242 cm^−1^ and 1127 cm^−1^, and the C-O vibration peak corresponded with a wavelength of 1022 cm^−1^. The basic properties are listed in Table 1.

3. Waterborne acrylic acid

**Figure 4:**
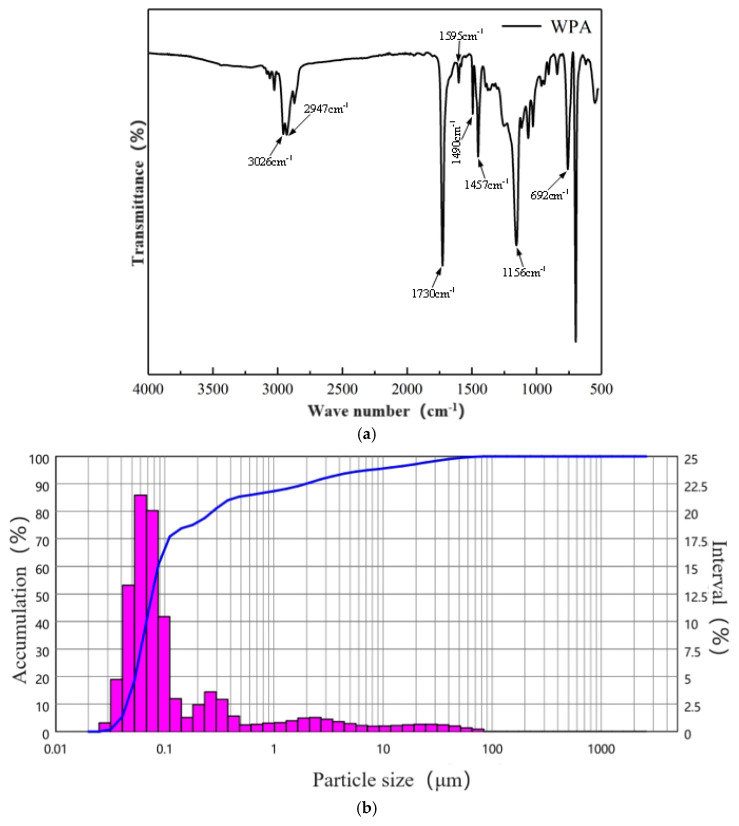
(**a**) Infrared spectrum of waterborne acrylic lotion. (**b**) Particle size curve of waterborne acrylic acid lotion.

The acrylic acid used in this test was a waterborne acrylic acid E0504 lotion produced by Shenzhen Jitian Chemical Co., Ltd., Shenzhen, China. The infrared spectrum obtained using a Brooke Vertex 70 (Bruker Optics Co., Ltd., Shanghai, China) is presented in Figure 4 (WPA: waterborne acrylic lotion). The stretching vibration peak of =C-H corresponded with a wavenumber of 3026 cm^−1^, the stretching vibration peak of -CH_2_- corresponded with a wavenumber of 2947 cm^−1^, the stretching vibration peak of C=O corresponded with a wavenumber of 1730 cm^−1^, the stretching vibration peak of the C=C double bond corresponded with wavenumbers of 1595 cm^−1^ and 1490 cm^−1^, the out-of-plane vibration peak of O-H corresponded with a wavenumber of 1457 cm^−1^, the stretching vibration peak of C-O corresponded with a wavenumber of 1156 cm^−1^, and wavenumbers 759 cm^−1^ and 692 cm^−1^ corresponded with the out-of-plane bending vibration peak of C-H. The basic properties are listed in Table 2.

4. Defoamer

Choosing a defoamer required the elimination of the adverse effects of polymer modification. Organic silicone polyether defoamers have good defoaming and defoaming properties, as well as low surface tension [17]. The defoamer used in our experiment was an organic silicon polyether-type defoamer produced at Huaxia Industrial Park (Wantong Road, Zaoyang City, Hubei Province). The defoamer was a slightly yellow liquid. The experimental dosage was 0.5% of the solid content of the waterborne polymers.

5. Silane coupling agent

Silane coupling agents have bifunctional properties that can react with inorganic fillers and polymer matrices, respectively, forming chemical bridging and improving the interfacial adhesion between the two. The alkoxy groups in silicon can be hydrolyzed into silanols, and reactive silanol groups can form hydrogen and chemical bonds with the polar groups (-OH) of water glass. It can also improve compatibility between inorganic fillers and polymer matrices, which is beneficial for achieving uniform filler distribution in the polymer matrix [18]. The silane coupling agent used in our experiment was KH-560 (the coupling agent KH-560 is based on 3−glycidoxypropyltrimethoxysilane), which was produced by Dongguan Zhongyu Wohao New Materials Co, Ltd., Dongguan, China. KH-560 has a colorless, transparent liquid appearance and is primarily used to improve the bonding performance of organic and inorganic materials. It is suitable for polymers such as epoxy, phenolic, polysulfide, polyurethane, and polystyrene. The experimental dosage was 1% of the solid content of the waterborne polymers.

### 2.2. Test Methods

#### 2.2.1. Basic Performance Test

We used the test method for the flowability of cement paste as defined in the reference specification GB/T8077-2012 (Test Method for Uniformity of Concrete Admixtures) [19]. The gelation time was measured using the inverted cup method. The physical properties of the dual-liquid slurry stone were used to primarily determine the compressive strength and flexural strength of the test block. The testing method was based on the national standard GB/T 17671-2021 (Test Method for Strength of Cement Mortar (ISO Method)) [20]. The experimental steps were as follows:

1. Pour the retarder into the water and stir for one minute to mix the water and retarder evenly. Then, pour the mixture into cement and stir until the two are evenly mixed.
2. Pour the silane coupling agent into the water-based lotion and stir for 1 min to mix the water-based lotion and silane coupling agent evenly.
3. Mix the above materials and add a defoamer, followed by stirring for three minutes.
4. Add the water-glass solution and stir quickly for 15 s.

#### 2.2.2. XRD Testing

A qualitative analysis was conducted using an X-ray diffractometer (TD-3500; Dandong Tongda Technology Co., Ltd., Dongguan, China). We selected the central sample of a standard specimen that had been cured for 28 days and ground it in a mortar to obtain a powder sample. This was dried in an oven at 45 °C for 24 h before testing. We used a scanning angle range of 15–80 °C with a step size of 0.02°. A graph analysis was performed using Jade 6.5 software. 

#### 2.2.3. Microstructure Testing

A SEM scanning instrument (German ZEISS Sigma 300, which was producted by CarlZeiss Vision Technologies (Guangzhou) Ltd., Guangzhou, China) was used to observe and analyze the stone specimens cured for 28 days. After the strength test was completed, we produced thin slices from the undamaged parts of the sample and dried them in an oven at 45 °C for 24 h to prepare a test sample. It was necessary to vacuum and spray gold onto the test sample prior to testing.

#### 2.2.4. Pore-S

The pore characteristics of the stone body were determined using a MesoMR12-060H-I-type nuclear magnetic resonance analysis system from Newmai Technology in Suzhou, China. We selected the part of the specimen that had been cured for 28 days and used a core-drilling machine to obtain samples. The core sample was placed in a -0.1 MPa vacuum saturation device for 24 h to obtain saturation. The sample was then placed into a low-field nuclear magnetic resonance instrument for testing. 

## 3. Comparative Analysis of Data

The 601R waterborne polyurethane, silane coupling agent, and defoamer were introduced to the commonly used dual-liquid slurry construction basic mix ratio. In the test, the water–cement ratio of the C-liquid was fixed at 0.8 (The material used in this experiment is for grouting and water blocking under rich water conditions. To extend the gelation time, avoid pipe blockage, and control the volume ratio of water glass, the water–cement ratio in this experiment is high). As the solid content of polyurethane lotion was 35%, the calculated water consumption in the water–cement ratio included the amounts of both the tap water and the water in the waterborne polyurethane lotion. The contents of waterborne polyurethane in the C-liquid were 0%, 2.5%, 5%, 7.5%, and 10% of the cement mass, respectively. The content of the silane coupling agent was fixed at 1% of the polymer solid content. The content of the defoamer was fixed at 0.5% of the polymer solid content. The specific experimental plan is presented in Table 3.

The amounts of acrylic acid in the acrylic-modified dual-liquid slurry were 0%, 2.5%, 5%, 7.5%, and 10% of the cement mass, respectively. As the solid content of the acrylic acid lotion was 48%, the calculated water consumption in the water–cement ratio included the amounts of both the tap water and the water in the water-based acrylic acid lotion. The mix design was basically consistent with that specified in Table 3. The specific experimental plan is presented in Table 4.

### 3.1. Fluidity

The experimental results of the effect of waterborne polyurethane on the flowability of cement–water-glass dual-liquid slurry according to the above experimental methods and mix designs are revealed in Table 5. The experimental results of the influence of waterborne acrylic acid on the flowability of cement–water-glass dual-liquid slurry are presented in Table 6.

**Figure 5:**
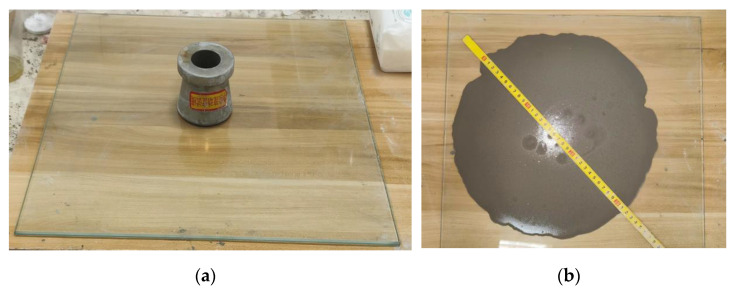
Fluidity detection: (**a**) glass plate and cone die; (**b**) dual-liquid slurry flow on a round surface.

The flowability of the modified dual-liquid slurry material decreased with an increase in the waterborne polyurethane content. The flowability of the modified dual-liquid slurry was almost unaffected by polyurethane in the content range of 0–2.5%, but the influence of a 5–10% content of polyurethane on the flowability of the modified dual-liquid slurry was significant. The fluidity of the dual-liquid slurry increased to a certain extent with an increase in the waterborne acrylic lotion content. This was because the acrylic acid lotion particles dispersed in the slurry to produce a “ball effect”, and the dispersion of surfactants in the lotion led to a water-reducing effect, improved fluidity, and easier construction [21,22]. Figure 5 shows the instrument and experimental schematic used for testing fluidity. The truncated cone circular mold in Figure 5a is the mold used for testing fluidity.

### 3.2. Gelation Time

The experimental results of the effect of adding polyurethane and acrylic acid in equal proportions on the flowability of a cement–water-glass dual slurry are shown in Table 7.

The gelation time of the modified dual-liquid slurry material gradually decreased as the content of waterborne polyurethane increased. This was similar to the effect of the polyurethane content on the fluidity of the modified dual-liquid slurry. We observed that the fluidity of the liquid rapidly decreased to the point of losing fluidity after adding 10% polyurethane to the C-liquid [23,24]. At this time, the C-liquid and S-liquid were mixed in a cup, but the two did not mix well. This resulted in an inability to determine the gelation time. The gelation time of the modified dual-liquid slurry material slightly increased with an increase in the waterborne acrylic lotion. The water-based acrylic acid lotion exhibited good bonding performance with the cement particles and was adsorbed on the surface of these particles. This delayed the reaction process between the cement slurry and water glass to an extent, leading to an extension of the gelation time of the dual-liquid slurry [25].

### 3.3. Physical Properties of Stones

**Figure 6:**
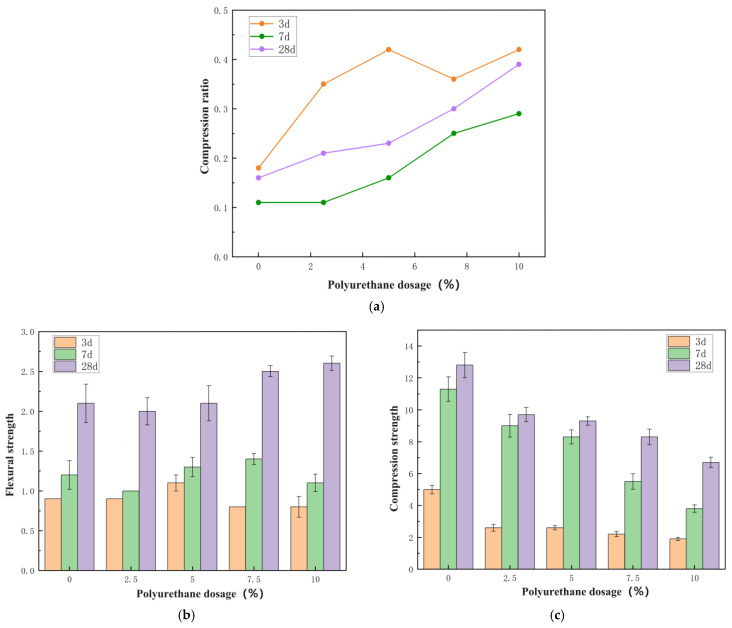
Effect of polyurethane content on the mechanical properties of the dual-liquid slurry: (**a**) compression ratio; (**b**) flexural strength; (**c**) compressive strength.

**Figure 7:**
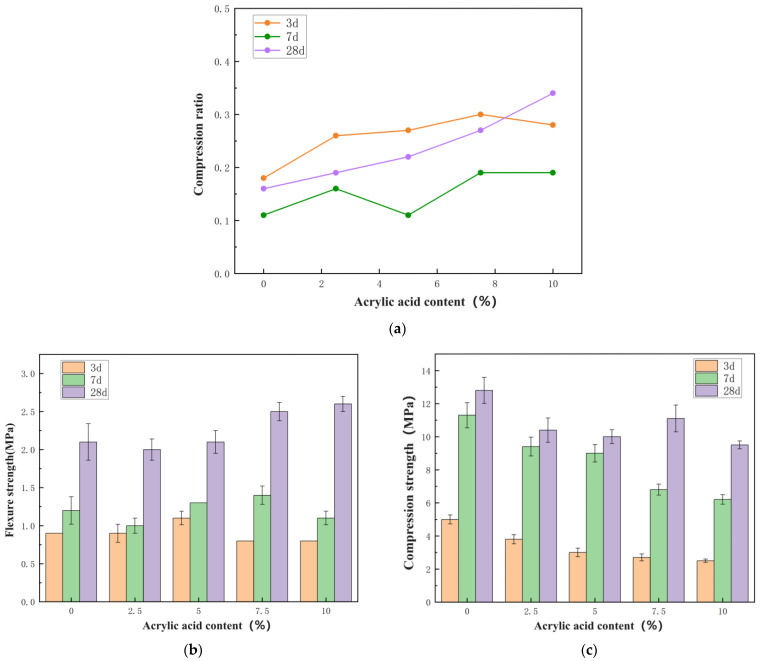
Effect of acrylic acid content on mechanical properties of dual-liquid slurry: (**a**) compression ratio; (**b**) flexural strength; (**c**) compressive strength.

The test pieces used for the mechanical-property test were prepared according to the mix proportions listed in Table 2 and Table 5. The compressive strength and flexural strength of the test pieces were measured over 3 d, 7 d, and 28 d, and the influence of the two types of polymer lotion on the physical properties of the dual-liquid slurry stones was analyzed. The test results of the mechanical properties of the polyurethane-modified dual-liquid slurry stones are presented in Table 8 and Figure 6, and the test results of the physical properties of the acrylic-modified dual-liquid slurry stones are revealed in Table 9 and Figure 7. The formula for calculating the compression ratio is as follows:

R = R_f_/R_c_

The following definitions apply in the above formula:

R—compression ratio, accurate to 0.1;
R_f_—flexural strength, MPa;
R_c_—compressive strength, MPa.

**Table 8:** Effect of polyurethane content on the mechanical properties of dual-liquid slurry.

Number	Flexural Strength (MPa)			Compressive Strength (MPa)			Compression Ratio		
	3 d	7 d	28 d	3 d	7 d	28 d	3 d	7 d	28 d
P0	0.9	1.2	2.1	5.0	11.3	12.8	0.18	0.11	0.16
PU1	0.9	1.0	2.0	2.6	9.0	9.7	0.35	0.11	0.21
PU2	1.1	1.3	2.1	2.6	8.3	9.3	0.42	0.16	0.23
PU3	0.8	1.4	2.5	2.2	5.5	8.3	0.36	0.25	0.30
PU4	0.8	1.1	2.6	1.9	3.8	6.7	0.42	0.29	0.39

**Table 9:** Effect of acrylic acid content on the mechanical properties of dual-liquid slurry.

Number	Flexural Strength (MPa)			Compressive Strength (MPa)			Compression Ratio		
	3 d	7 d	28 d	3 d	7 d	28 d	3 d	7 d	28 d
P0	0.9	1.2	2.1	5.0	11.3	12.8	0.18	0.11	0.16
PA1	1.0	1.5	2.0	3.8	9.4	10.4	0.26	0.16	0.19
PA2	0.8	1.0	2.2	3.0	9.0	10.0	0.27	0.11	0.22
PA3	0.8	1.3	3	2.7	6.8	11.1	0.30	0.19	0.27
PA4	0.7	1.2	3.2	2.5	6.2	9.5	0.28	0.19	0.34

Water-based polyurethane demonstrated a slight increase in the flexural strength of the C-S dual-liquid slurry within a certain dosage, but the compressive strength of the stone body in all dual-liquid slurries doped with polyurethane significantly decreased. The flexural compression ratio increased to a certain extent. The main developmental period of the flexural strength of the polyurethane-modified dual-liquid slurry stone body occurred from 7 d to 28 d, and the main developmental period of compressive strength was from 3 d to 7 d. Waterborne polyurethane had a negative impact on the overall physical properties of the stones.

Water-based acrylic acid improved the flexural strength of the C-S dual-liquid slurry within a certain dosage to a certain degree, but the compressive strength of the stone bodies of all dual-liquid slurries mixed with polyurethane continued to demonstrate a significant decrease. The flexural compression ratio had a certain increase. The main developmental period of the flexural strength of the acrylic-modified dual-liquid slurry stone body occurred from 7 d to 28 d, and the main developmental period of compressive strength was from 3 d to 7 d. Waterborne acrylic acid had a negative impact on the overall physical properties of the stones, primarily because the introduction of acrylic acid hindered the cement hydration reaction and the generation of hydration products. This was similar to the effect of polyurethane on the dual-liquid slurry. However, when comparing the compressive strength of 28-day polyurethane-modified double-liquid slurry stone bodies, it can be seen that at the same dosage, the negative impact of acrylic acid on the compressive strength of double-liquid slurry stone bodies is less than that of polyurethane.

**Figure 8:**
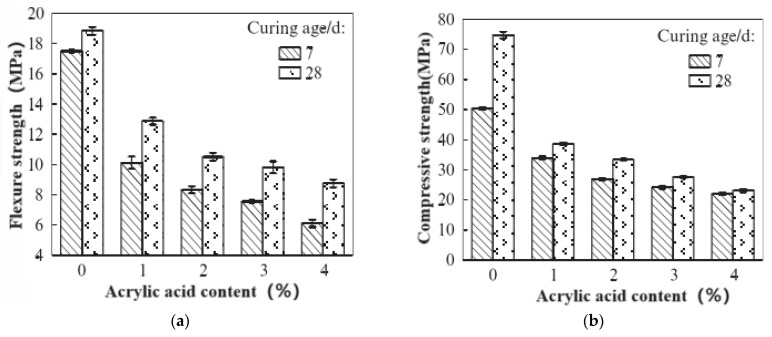
Mechanical properties of acrylic-acid-modified MOC (**a**) flexural strength; (**b**) compressive strength [26].

Some scholars have also studied the properties of acrylic-acid-modified MOC (magnesium oxychloride cement); however, comparing Figure 7 and Figure 8, it can clearly be seen that the flexural strength of C-S dual liquid slurry increases with an increase in acrylic acid, and the compressive strength fluctuates but does not decrease much after 28 days. However, magnesium oxychloride cement decreases linearly after the addition of acrylic acid, indicating that acrylic acid is more suitable for a dual-liquid slurry.

## 4. Characterization Analyses

### 4.1. XRD

**Figure 9:**
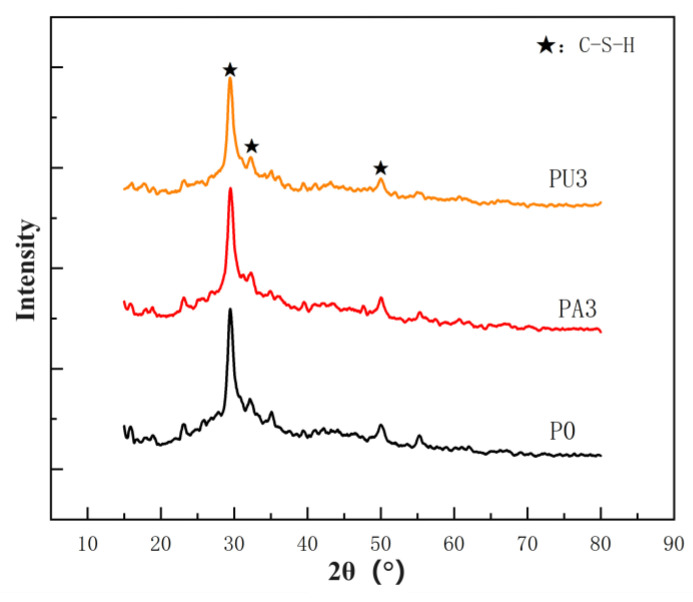
XRD patterns of dual-fluid slurry stone body.

Figure 9 displays the XRD image of a 28-day-old dual-fluid slurry stone body. An analysis using Jade 6.5 software revealed that the main hydration product was hydrated calcium silicate. The graph indicated that waterborne acrylic acid and waterborne polyurethane did not alter the types of hydration products.

No characteristic peak of Ca(OH)_2_ was observed in the hydration product, likely because of the introduction of a large amount of water glass into the mix proportion of the dual-liquid slurry stone body. The reaction between the water glass and the cement slurry consumed the Ca(OH)_2_ generated in the hydration process, which further reacted to form a C-S-H gel. Certain scholars have elaborated on the relationship between Ca (OH)_2_ and C-S-H [27]. As the Ca(OH)_2_ was dissolved in C-S-H, the Ca(OH)_2_ was saturated and precipitated during the dual-liquid slurry reaction, forming a solid solution structure. A significant amount of calcium hydroxide may have been extracted through the chemical composition of the C-S-H gel. No obvious characteristic peak of ettringite was observed because of the poor crystallinity of the main hydration product and the C-S-H gel. Thus, less ettringite was generated by the hydration reaction. Additionally, the intensity of this diffraction peak is relatively low, and no other peaks were identified as obvious diffraction peaks of other crystals.

### 4.2. Microstructure

**Figure 10:**
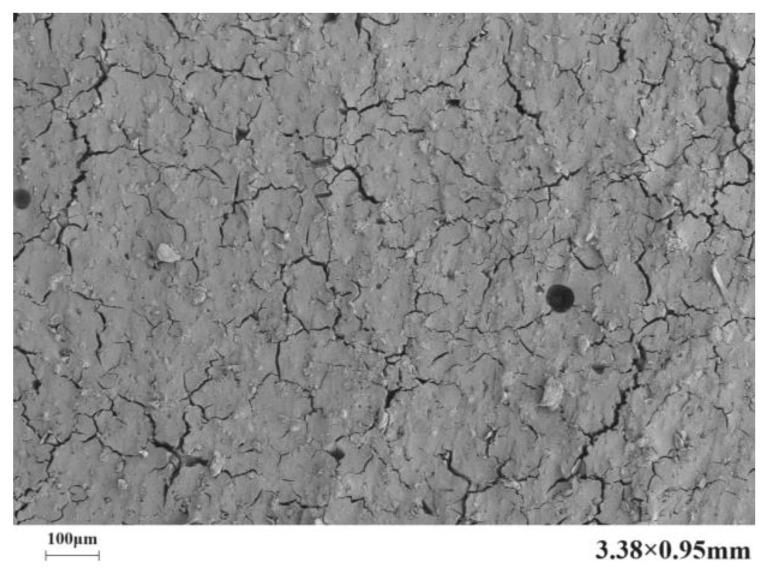
Scanning electron microscopy image of P0 stone body magnified 200×.

**Figure 11:**
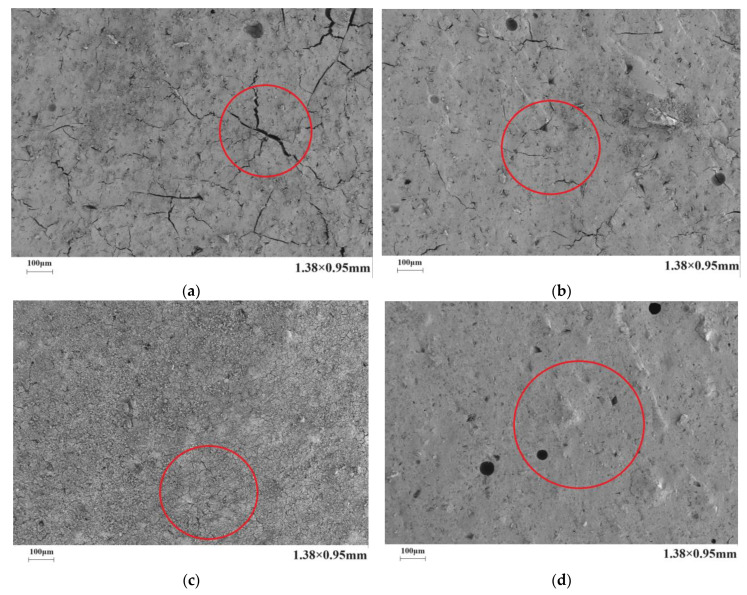
Scanning electron microscopy images of stone bodies with different polyurethane dosages: (**a**) PU1 magnified 200×; (**b**) PU2 magnified 200×; (**c**) PU3 magnified 200×; (**d**) PU4 magnified 200×.

**Figure 12:**
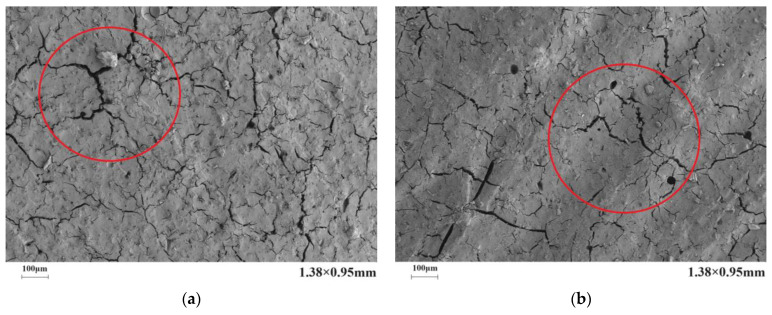

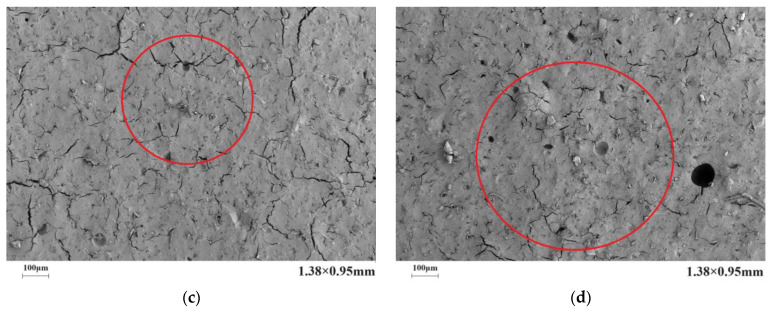
Scanning electron microscopy images of stone bodies with different acrylic acid dosages: (**a**) PA1 magnified 200×; (**b**) PA2 magnified 200×; (**c**) PA3 magnified 200×; (**d**) PA4 magnified 200×.

**Figure 13:**
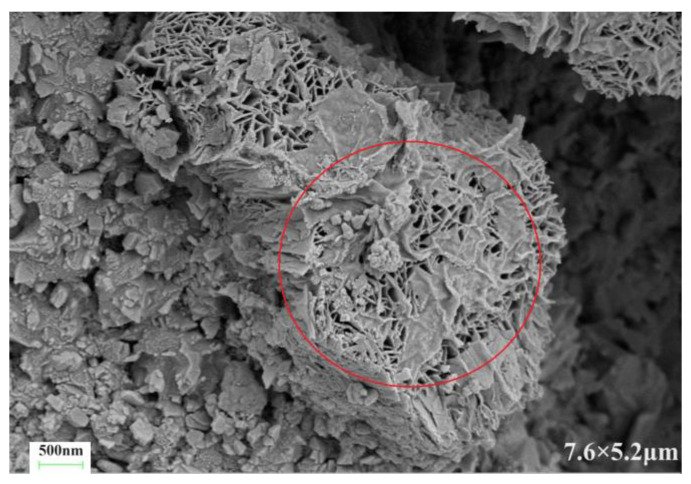
Scanning electron microscopy image of P0 stone body magnified 40,000 times.

**Figure 14:**
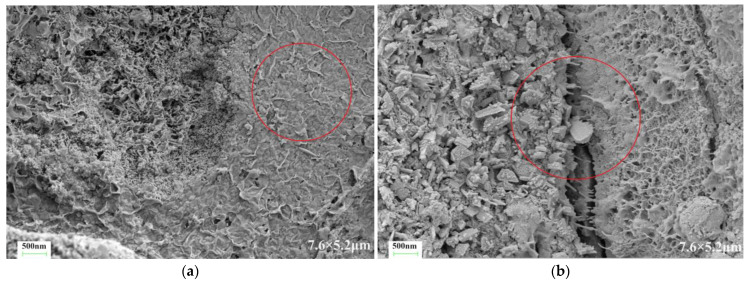
Scanning electron microscopy images of PU3 and PA3 stone bodies magnified 40,000 times: (**a**) PU3 magnified 40,000 times; (**b**) PA3 magnified 40,000 times.

Figure 10 presents the microstructure of the C-S dual-liquid slurry without a polymer addition. Figure 11a–d reveal the microstructure of the modified C-S dual-liquid slurry with polyurethane contents of 2.5–10%, respectively. The samples were prepared according to the mix proportion listed in Table 3. Figure 12a–d reveal the microstructure of the modified C-S dual-liquid slurry with acrylic acid contents of 2.5–10%. Figure 13 presents the microstructure magnified 40,000 times for P0, and Figure 14 presents the microstructure magnified 40,000 times for PU3 and PA3. The samples were prepared according to the mix proportion listed in Table 4, with a curing period of 28 days. The test samples were thin layers inside the stone body. 

The generated stone body was prone to cracking because of the high water–cement ratio of the C-S dual-liquid slurry material. The dehydration of the sample led to the formation of cracks during the sample preparation process, resulting in changes in the microstructures of the test sample and the curing sample.

Figure 11a–d reveal the cross-sectional views of the dual-liquid slurry stones with polyurethane contents of 2.5–10%. It can be clearly seen from the red marked sections in the figures that the number of cracks on the cross-section of the stone body decreased as the polyurethane content increased, and the width and length of the cracks both decreased. Figure 12a–d present the cross-sectional views of the dual-liquid slurry stones with acrylic acid contents of 2.5–10%. We can see from the red marked area that the number, width, and length of cracks on the cross-section of the stones decreased as the acrylic acid content increased, but the effect was not significant. When the dosage of acrylic acid was small, the improvements in the cross-sectional structure of the stone body were not significant or even had a negative impact. When the dosage reached a certain level, it effectively improved the microstructure of the dual-liquid slurry stone body, reduced the generation of cracks during the dehydration process, and enhanced the crack-resistance performance of the stone body.

The red marked part in Figure 13 displays the morphological characteristics of the hydration products of a traditional dual-liquid slurry stone body. C-S-H gels with different shapes can be observed. The structure of the hydration products was loose and cracks were obvious. Kalina et al. [28] observed that the shape of a gel is related to its aging. Young gel has a smoother texture, whereas older gel usually crystallizes into a petal shape. Ca/Si has a direct impact on the morphology and structure of C-S-H gels. When Ca/Si is small, the C-S-H gel is lamellar and the spacing between lamellas is small. With an increase in Ca/Si, the lamellar morphology of C-S-H gels gradually disintegrates into a needle-bar shape and the structure changes from dense to loose [29].

A clear polyurethane film-forming effect can be observed in the left image of Figure 14. From the red marked parts in the figure, it can be seen that a dense and uniform polyurethane film is covered on the hydration productThe polyurethane mesh structure on the left of the image filled the pores of the hydration product, interweaving and tightly bonding with the hydration product [30,31]. Compared with the traditional dual-liquid slurry stone structure, the polyurethane-modified dual-liquid slurry stone structure was denser. The right figure in Figure 14 displays the microscopic morphology of acrylic-acid-modified dual-liquid slurry stones. Acrylic acid adsorbed on the surface of the hydration products and formed a polymer film. A clear salt bridge can be seen in the red marked part in the figure, filling the gaps between the hydration products, enhancing the bonding between interfaces, and inhibiting the development of cracks [32,33]. The addition of acrylic acid reduced the formation of cracks and compacted the structure of the stone body, but the effect was weaker than with polyurethane. Compared with the film-forming effect of polyurethane, the film-forming effect of acrylic acid was not significant and revealed a smaller inhibitory effect on the hydration products [34].

No other obvious characteristics in the cement hydration products were observed in Figure 12 and Figure 13.

### 4.3. Pore Distribution

**Figure 15:**
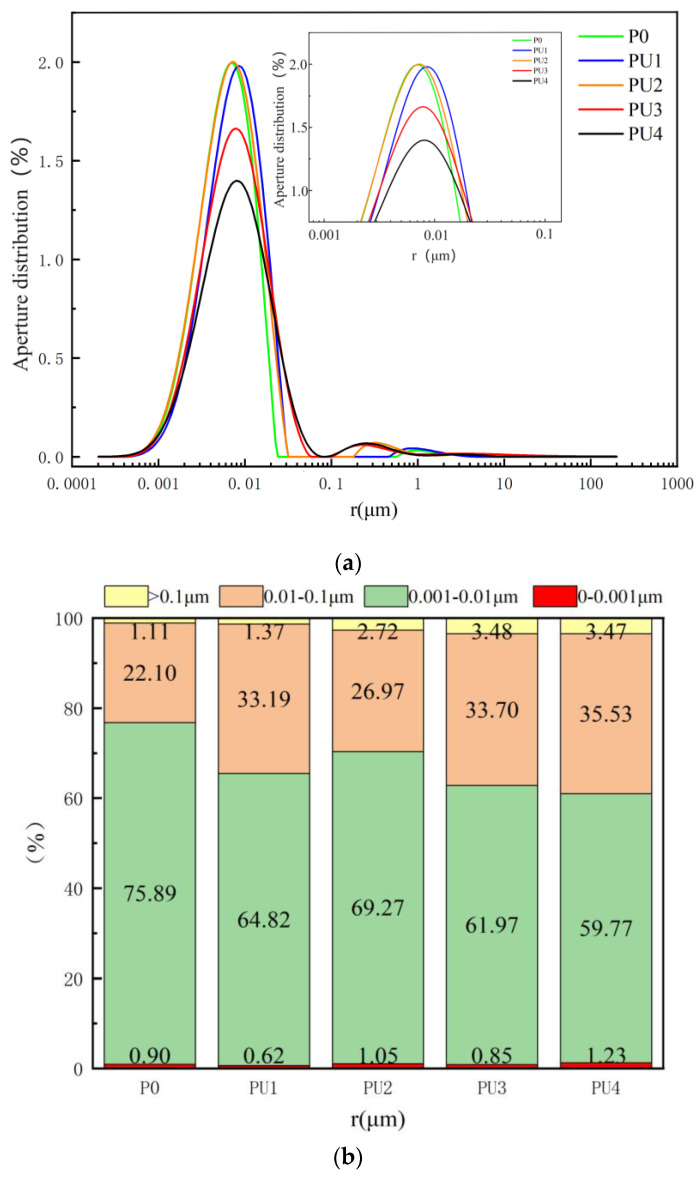
Pores-size distribution of stone bodies with different polyurethane dosages: (**a**) aperture distribution curve; (**b**) aperture distribution ratio.

Figure 15 presents the pore-size distribution of the stone bodies with different polyurethane contents. The test specimens were prepared according to the mix proportion listed in Table 3, and the curing age was 28 days. Figure 9 presents the pore-size distribution of the stone bodies with different amounts of acrylic acid. The test specimens were prepared according to the mix proportion listed in Table 4, and the curing age was 28 days. Figure 15a reveals that the porosity remained almost unchanged at polyurethane contents of 2.5% and 5% but decreased at 7.5% and 10%. The smallest porosity was observed at 10%. Figure 15b reveals that the pore-size distribution of the polyurethane-modified C-S dual-liquid slurry stones was primarily between 0.001 and 0.1 μm, accounting for over 90%. There was no significant linear change in the pore ratio in the 0–0.001 μm range of the stone body with an increase in the polyurethane content. The introduction of polyurethane did not fundamentally affect the generation of pores in this range.

**Figure 16:**
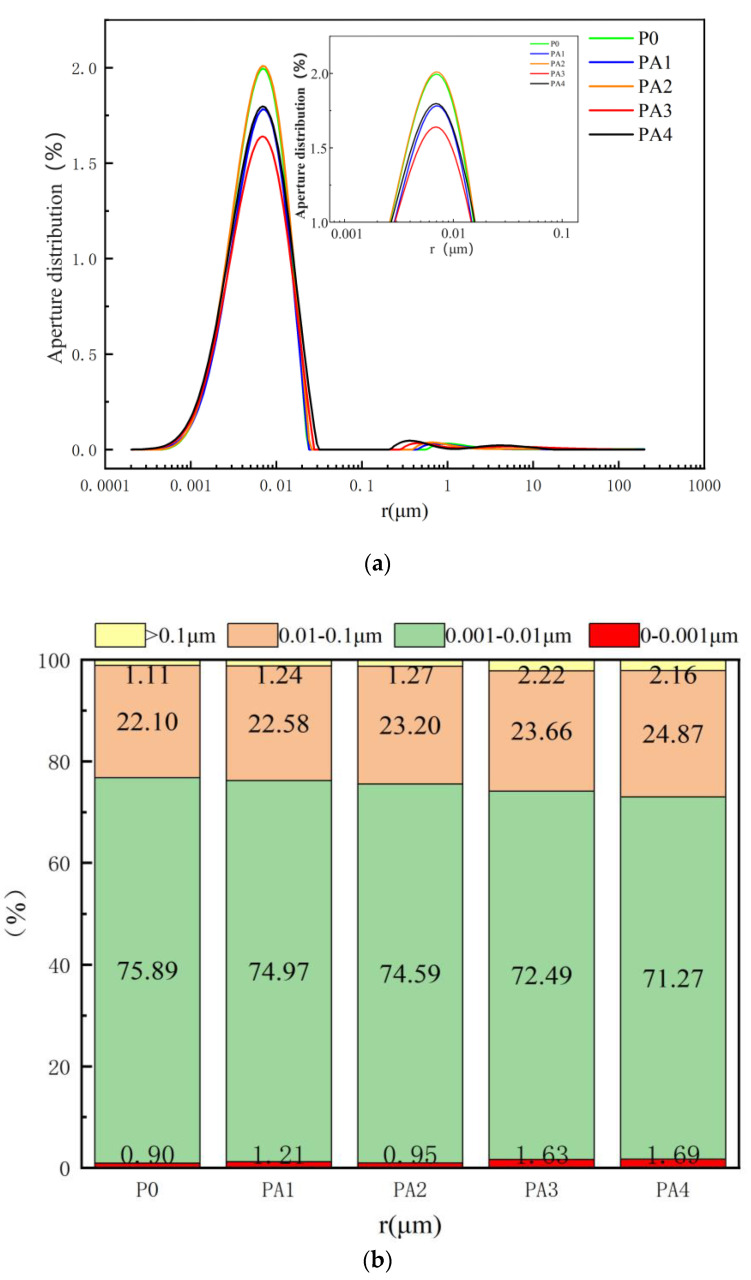
Pores-size distribution of stone bodies with different acrylic acid dosages: (**a**) aperture distribution curve; (**b**) aperture distribution ratio.

Figure 16a reveals that the porosity remained almost unchanged at acrylic acid contents of 2.5% and 5% but decreased at 7.5% and 10%. The smallest porosity was observed at 7.5%. Figure 16b reveals that the pore-size distribution of the acrylic-acid-modified C-S dual-liquid slurry stones was primarily between 0.001 and 0.1 μm, accounting for over 90%. The porosity and dense structure of the stone body were reduced with an increase in the acrylic acid content, but the proportion of generated pores > 0.01 μm increased. The small peak of the original pore-size distribution around 1 μm shifted to 0.1–1 μm, and the peak value increased. This was the same as the influence of the water-based polyurethane lotion on the pore distribution of the dual-liquid slurry stone body, but the influence degree was significantly smaller.

## 5. Conclusions 

In this study, we modified a traditional C-S dual-liquid slurry material by introducing a waterborne polyurethane lotion and a waterborne acrylic lotion. The mixture ratio of the C-S dual-liquid slurry was adjusted, and the basic properties of the dual-liquid slurry were tested. The material properties were characterized using X-ray diffraction analyses, SEM observations, and a nuclear magnetic resonance test, which provided effective support to determine a specific modified mixture ratio. The results provided the following conclusions:

1. The aqueous polyurethane lotion reduced the fluidity of the dual-liquid slurry, while the waterborne acrylic lotion improved the fluidity of the liquid, increasing the fluidity of the dual-liquid slurry.
2. The higher the content of waterborne polyurethane lotion, the shorter the gelation time of the dual-liquid slurry. The higher the content of waterborne acrylic lotion, the longer the gelation time of the dual-liquid slurry.
3. Waterborne polyurethane lotion and waterborne acrylic lotion provided a small increase in the flexural strength of a C-S dual-liquid slurry at a certain amount, but the compressive strength of all dual-liquid slurry stones mixed with polyurethane and acrylic acid significantly decreased. The flexural compression ratio demonstrated a certain increase.
4. The main chemical substance of the dual-liquid slurry stone body was hydrated calcium silicate. Waterborne acrylic acid and waterborne polyurethane did not change the types of hydration products.
5. The introduction of polyurethane and acrylic acid during the dehydration process effectively reduced the number, width, and length of cracks in the microstructure of a stone body. The improvement effect of polyurethane on the microstructure of a stone body was significantly better than that of acrylic acid.
6. The introduction of both polyurethane and acrylic acid increased the proportion of pores > 0.01 μm generated in a stone body, but the degree of influence varied. The influence of acrylic acid on the pore distribution of the stone body was lower than that of polyurethane. Although polyurethane significantly improved the crack resistance of stone bodies during the dehydration process and the resulting porosity was low when the dosage was high, polyurethane had adverse effects on the flowability and gelation-time performance of the dual-liquid slurry. The flowability and gelation-time performance of the dual-liquid slurry were excellent when the acrylic acid dosage was 7.5%. The compressive strength of the stone body slightly decreased, the flexural strength improved to a certain extent, the crack resistance of the stone body was also enhanced during the dehydration process, and the porosity of the stone body was low. Therefore, the optimal dosage was determined to be 7.5%.

## Figures and Tables

**Table 1:** Basic properties of waterborne polyurethane.

Type	Appearance	Solid Content	PH Value
Anionic	Transparent liquid	35%	7–9

**Table 2:** Basic properties of waterborne acrylic lotion.

Type	Appearance	Solid Content	PH Value
Anionic	Transparent liquid	48%	5–6

**Table 3:** Mix proportions of polyurethane-modified cement–water-glass dual-liquid slurry.

Number	Water–Cement Ratio	Polyurethane (%)	Coupling Agent (%)	Defoamer (%)	Original C:S Volume Ratio
P0	0.8	0	0	0	1:0.6
PU1	0.8	2.5	1%	0.5	1:0.6
PU2	0.8	5	1%	0.5	1:0.6
PU3	0.8	7.5	1%	0.5	1:0.6
PU4	0.8	10	1%	0.5	1:0.6

**Table 4:** Mix proportions of acrylic acid-modified cement–water-glass dual-liquid slurry.

Number	Water–Cement Ratio	Acrylic Acid (%)	Coupling Agent (%)	Defoamer (%)	Original C:S Volume Ratio
P0	0.8	0	0	0	1:0.6
PA1	0.8	2.5	1	0.5	1:0.6
PA2	0.8	5	1	0.5	1:0.6
PA3	0.8	7.5	1	0.5	1:0.6
PA4	0.8	10	1	0.5	1:0.6

**Table 5:** Effect of polyurethane content on the flowability performance of dual-liquid slurry.

Polyurethane Dosage (%)	Fluidity (mm)
0	323
2.5	325
5	305
7.5	278
10	240

**Table 6:** Effect of acrylic acid content on the flowability performance of dual-liquid slurry.

Acrylic Acid Content (%)	Fluidity (mm)
0	323
2.5	320
5	325
7.5	330
10	326

**Table 7:** The influence of polyurethane and acrylic acid on the gelation time of dual-liquid slurry.

Dosage (%)	Gelation Time after Adding Polyurethane (s)	Gelation Time after Adding
		Acrylic Acid (s)
0	58	58
2.5	57	60
5	51	61
7.5	47	62
10	—	66

## Data Availability

The data presented in this study are available on request from the corresponding author. The data are not publicly available due to patent applications.
